# Clinical implementation of simultaneous multiple biomarkers testing for metastatic or recurrent gastroesophageal adenocarcinoma: a single-institutional experience

**DOI:** 10.1016/j.esmogo.2024.100086

**Published:** 2024-08-02

**Authors:** U. Okazaki, I. Nakayama, N. Sakamoto, T. Kuwata, A. Kawazoe, M. Yoshida, M. Yura, Y. Matsubara, A. Jubashi, S. Sato, S. Ushiyama, Y. Miyashita, A. Kobayashi, T. Hashimoto, S. Mishima, D. Kotani, Y. Nakamura, Y. Kuboki, H. Bando, T. Kojima, T. Yoshino, T. Kinoshita, K. Shitara

**Affiliations:** 1 Department of Gastroenterology and Gastrointestinal Oncology, National Cancer Center Hospital East, Kashiwa, Chiba, Japan; 2 Department of Pathology and Clinical Laboratories, National Cancer Center Hospital East, Kashiwa, Chiba, Japan; 3 Department of Genetic Medicine and Services, National Cancer Center Hospital East, Kashiwa, Chiba, Japan; 4 Department of Gastric Surgery, National Cancer Center Hospital East, Kashiwa, Chiba, Japan

**Keywords:** gastroesophageal adenocarcinoma, immunohistochemistry, biomarker, personalized treatment, clinical practice

## Abstract

**Background:**

Evaluating multiple biomarkers including human epidermal growth factor receptor 2 (HER2), mismatch repair (MMR) status, programed death-ligand 1 (PD-L1), and claudin 18.2 (CLDN18.2) is essential for selecting appropriate first-line therapy of metastatic gastroesophageal adenocarcinoma (mGEA). However, this can be challenging if tumor tissue amount is limited and may cause delays in the initiation of chemotherapy. Therefore, we assessed the feasibility of multiple biomarkers testing in routine clinical practice.

**Materials and methods:**

We reviewed the medical records of treatment-naïve patients with mGEA who underwent prospective multiple biomarkers testing between April 2022 and October 2023 in our institution. Eight biomarker status including HER2, MMR, PD-L1, CLDN18.2, Epstein–Barr virus, fibroblast growth factor receptor 2, epidermal growth factor receptor and mesenchymal epithelial transition expressions were simultaneously examined using biopsy or surgical specimens.

**Results:**

A total of 203 patients with mGEA were analyzed. Most patients underwent gastroendoscopy and tumor biopsy shortly after referral to our institution. Biomarkers testing was successfully completed on the first attempt in 198 patients (97.5%). With additional steps including additional biopsy or asking remaining tumor samples from the referring hospital, the biomarker results were eventually available in all cases. The median turnaround time from sample collection to reporting results was 7 days. Consequently, 178 patients (87.7%) could receive first-line treatment after obtaining the biomarker status.

**Conclusions:**

Multiple biomarkers testing for patients with mGEA was feasible in clinical practice. Establishment of a testing infrastructure based on the collaboration with multiple departments is essential for implementing biomarker-driven precision treatment.

## Highlights

• Multiple biomarkers testing is feasible in routine clinical practice mainly using biopsy samples.
• Median turnaround time for reporting biomarker results was 7 days.
• Approximately 90% patients started first-line systemic chemotherapy in consideration with biomarker status.

## Introduction

Gastroesophageal adenocarcinoma (GEA) is the fifth most common cancer in the world and the fourth leading cause of cancer-related deaths worldwide.^1^ GEA incidence and mortality rates are particularly high in East Asia, Eastern Europe, and South America. Systemic chemotherapy is the standard of care for patients with metastatic advanced or recurrent GEA (mGEA).^2^, ^3^, ^4^, ^5^ Several clinical trials have successfully integrated the targeted therapy and immunotherapy into first-line chemotherapy for patients with mGEA.

Anti-human epidermal growth factor receptor 2 (HER2) monoclonal antibody, trastuzumab, plus chemotherapy has been standard for HER2-positive disease.^6^ Adding the anti-programmed cell death 1 (PD-1) pembrolizumab further improved treatment outcomes, especially for patients with HER2-positive and programmed death-ligand1 (PD-L1) combined positive score (CPS) of 1 or higher.^7^ For HER2-negative disease, PD-1 blockade with first-line chemotherapy prolonged overall survival (OS), particular in patients with higher CPS or deficient mismatch repair (dMMR).^8^, ^9^, ^10^ Recently, zolbetuximab, a claudin 18.2 (CLDN18.2)-targeted monoclonal antibody, in combination with first-line chemotherapy has also improved OS of patients with HER2-negative and CLDN18.2-positive mGEA.^11^^,^^12^ First-line treatment options for patients with mGEA vary based on biomarkers: HER2-positive cases may include trastuzumab, plus pembrolizumab added if CPS is 1 or higher. In HER2-negative, nivolumab or pembrolizumab according to PD-L1 CPS, or zolbetuximab for CLDN18-positive cases, are the other standard. Additionally, while rare, dMMR is a predictive factor independent of HER2 status, warranting preferential use of nivolumab or pembrolizumab. This complicated biomarker-based personalized approach reflects the current landscape.

Biomarkers testing for HER2, PD-L1, MMR and CLDN18.2 is necessary for patient selection in first-line treatment of patients with mGEA. However, this can be challenging if the tumor tissue sample is limited and may cause potential delays in the initiation of chemotherapy. Actual feasibility of simultaneous multiple biomarkers testing in routine clinical practice remains unknown. Herein, we present a single-institutional experience of prospective multiple biomarkers testing before first-line chemotherapy for mGEA.

## Materials and methods

### Patients

The purpose of this analysis was to evaluate the feasibility of simultaneous multiple biomarkers testing for patient selection of first-line treatment of mGEA at the National Cancer Center Hospital East. We reviewed the medical records of consecutive treatment-naïve patients who had received prospective multiple biomarkers testing for HER2, MMR proteins [MutL Homolog 1 (MLH1), MutS Homolog 2 (MSH2), MutS Homolog 6 (MSH6), and Postmeiotic Segregation Increased 2 (PMS2)], PD-L1, and CLDN18 at our institute between April 2022 and October 2023. During this period, we also tested additional exploratory biomarkers including Epstein–Barr virus (EBV), expression of fibroblast growth factor receptor 2 (FGFR2), epidermal growth factor receptor (EGFR), and mesenchymal epithelial transition (MET) as previously reported.^13^^,^^14^ Therefore, in total, eight biomarkers were tested.

The patients who met the following eligibility criteria were analyzed in this study: (i) histologically proven GEA, (ii) unresectable advanced or recurrent GEA, (iii) previously untreated with systemic chemotherapy for metastatic disease, and (iv) provided written informed consent with participation to biomarker study (UMIN000019129). We excluded the patients who did not receive chemotherapy at our institute. The biomarker study protocol was approved by the ethics committee of our institution, National Cancer Center Hospital East Certified Review Board, and was conducted in accordance with the guidelines for biomedical research stipulated in the Declaration of Helsinki.

Most patients underwent gastroendoscopy and tumor biopsy shortly after referral to our institution. A total of six biopsy samples were taken during each endoscopic procedure. In the case of recurrent gastric cancer after gastrectomy, we conducted biomarkers testing using surgical materials. If the initial tumor sample had insufficient tumor content and deemed inadequate, an additional tumor biopsy was carried out, or we requested remaining tumor samples from the referring hospital and repeated biomarkers testing.

### Biomarkers testing and assessment

Multiple biomarkers testing was conducted using available formalin-fixed paraffin-embedded (FFPE) tissue specimens. Immunohistochemistry (IHC) of HER2 was carried out with a monoclonal anti-HER2 antibody [PATHWAY HER2 (4B5), Ventana, Tucson, AZ] and fluorescence *in situ* hybridization (FISH) was assessed with the PathVysion HER-2 Probe Kit (Abbott Laboratories, Abbott Park, IL) for only IHC 2+. HER2 positivity was defined as IHC 3 + or IHC 2+ and FISH positive. The expressions of MMR proteins were evaluated using monoclonal antibodies for anti-MLH1 (ES05), anti-MSH2 (FE11), anti-PMS2 (EP51), and anti-MSH6 (EP49) (Agilent Technologies, Santa Clara, CA). Complete loss of MLH1, MSH2, PMS2, or MSH6 expression was considered MMR deficient (dMMR), whereas tumors that maintained MLH1, MSH2, PMS2, and MSH6 expressions were considered MMR proficient (pMMR). PD-L1 expression was assessed using an anti-PD-L1 rabbit monoclonal antibody (22C3 or 28-8 pharmDx assay). PD-L1 expression level was measured by CPS, defined as the ratio of the number of PD-L1-positive cells (tumor cells, lymphocytes, and macrophages) to the total number of tumor cells multiplied by 100. CLDN18.2 expression was evaluated with 43-14A clone (Roche Ventana, Oro Valley, AZ). CLDN18.2 positive was defined as moderate-to-strong expression in ≥75% of tumor cells, consistent with the criteria adopted in the SPOTLIGHT and GLOW trials. Chromogenic *in situ* hybridization for EBV-encoded RNA (EBER) was carried out using fluorescein-labeled oligonucleotide probes to assess the EBV status, while the antibodies for other biomarkers such as EGFR (3C6; Ventana), FGFR2 (K-sam; IBL), and MET (SP44; Ventana). The positivity of EGFR, FGFR2, and MET was defined as moderate-to-strong membrane staining in ≥50%, ≥10%, and ≥1% of tumor cells, respectively, as per previous reports.^13^^,^^14^ As a result, in total, 11 FFPE slides were needed to complete all planned eight biomarkers analyses.

All pathological specimens were reviewed by two board-certificated pathologists (NS and TK).

Turnaround time (TAT) of biomarkers analysis was defined as the interval between the first tumor specimen collection and reporting of the results.

## Results

### Patient characteristics

A total of 459 patients were enrolled in the biomarker study between April 2022 and October 2023. Among them, 181 patients with resectable locally advanced GEA were excluded, leaving 278 patients with mGEA. Furthermore, 57 patients who had received systemic chemotherapy before enrolling in the biomarker study and an additional 18 patients who did not receive any systemic chemotherapy at our institute were excluded from the analysis. Ultimately, 203 patients were eligible for this analysis (Supplementary Figure S1, available at https://doi.org/10.1016/j.esmogo.2024.100086).

The patient characteristics were listed in Table 1. Median age was 69 years (range 27-88 years), with 139 patients (68.5%) being male. Macroscopic type 4 tumor accounted for 26.6% and gastroesophageal junction (GEJ) primary was observed in 30.5%. One hundred and seventy-two (84.7%) patients had mGEA at the initial presentation and the remaining 31 patients (15.3%) had recurrent disease after previous gastrectomy.

**Table 1:** Baseline patient characteristics (*n* = 203)

Characteristics	*n*, (%)
Gender	
Male	139 (68.5)
Female	64 (31.5)
Age, median (range)	69 (27-88)
Tumor location	
GEJ	62 (30.5)
Stomach	141 (69.5)
Borrmann type	
Type 4	54 (26.6)
Non-type4	145 (71.4)
NA	4 (2.0)
Lauren’s type	
Diffuse	144 (70.9)
Intestinal	54 (26.6)
Unclassified	5 (2.5)
Extent of disease	
Metastatic	172 (84.7)
Recurrent	31 (15.3)
No. of metastases	
2>	95 (46.8)
2≤	108 (53.2)
Metastases site	
Liver	49 (24.1)
Lymph node	103 (50.7)
Peritoneum	90 (44.3)

GEJ, gastroesophageal junction; NA, not assesable; No, number.

### Tumor sample collection

Among 179 patients with metastatic disease at the initial presentation, all patients underwent endoscopic biopsy. Tumor samples were inadequate in five patients, but alternative tumor samples were available in all five patients after re-biopsy or requesting samples to the referring hospital. Of 24 patients (11.8%) with recurrent disease after gastrectomy, samples from baseline biopsy or resected tumors in our institution were used in 13 patients. Tumor biopsy for recurrent disease was carried out in five patients. Liver metastasis was utilized in two patients and, bone metastasis, peritoneum dissemination, and local recurrence were each found in a patient. Tumor samples were provided from the referring hospital for the remaining six patients. As a result, biopsy samples from the primary tumor site were assessed in 188 cases (92.6%), surgical samples of primary tumor in 10 cases (4.9%), and biopsy from metastasis/recurrent disease were used in 5 cases (2.5%). In 192 cases (94.6%), samples collected at our institute were assessed.

### Feasibility of multiple biomarkes testing

Multiple biomarkers testing was successfully completed on the first attempt in 198 patients (97.5%). Adenocarcinoma was not detected in the biopsy specimen collected at out hospital in the remaining five patients and other samples were retrieved from previous hospitals. Out of these five patients, three presented with type 4 gastric cancer. Therefore, the success rate of multiple biomarker assessment on the first attempt was slightly lower in patients with type 4 gastric cancer (94.4%) than that of non-type 4 mGEA (98.7%) (Supplementary Figure S2, available at https://doi.org/10.1016/j.esmogo.2024.100086). Eventually, biomarker results were available in all 203 patients, and the success rate was 100%. Success rates using surgical samples of the primary site (*n* = 10) and biopsy samples of metastatic/recurrent disease (*n* = 5) were both 100%.

### Turnaround time

**Figure 1:**
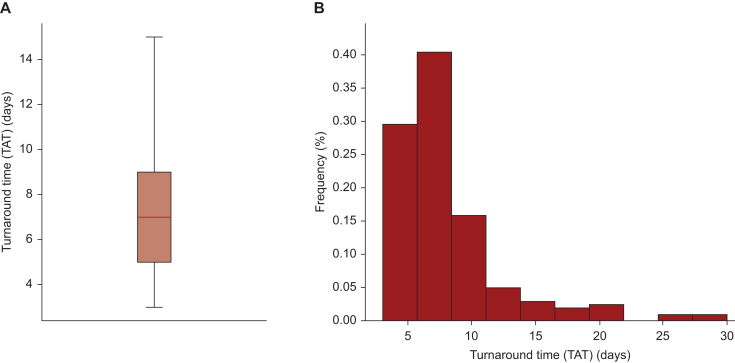
**Turnaround time**. (A) Box plot representing the TAT distribution. The bold line displays the median TAT and thin lines the 10th and 90th percentiles, indicating the lower and upper bounds of the data distribution, respectively. (B) The histogram illustrates the frequency distribution of TAT values. TAT, turnaround time.

Median TAT was 7 days (range 3-30 days) (Figure 1A). The results were obtained within 14 days in 187 cases (92.1%) (Figure 1B). Limited to the cases (*n* = 192) where the samples were collected at our own institution, the median TAT was 7 days (range 3-26 days). In five cases where adenocarcinoma was not detected on the first biopsy samples, TATs were 16, 21, 26, 28, and 30 days, respectively.

### Biomarker results

**Figure 2:**
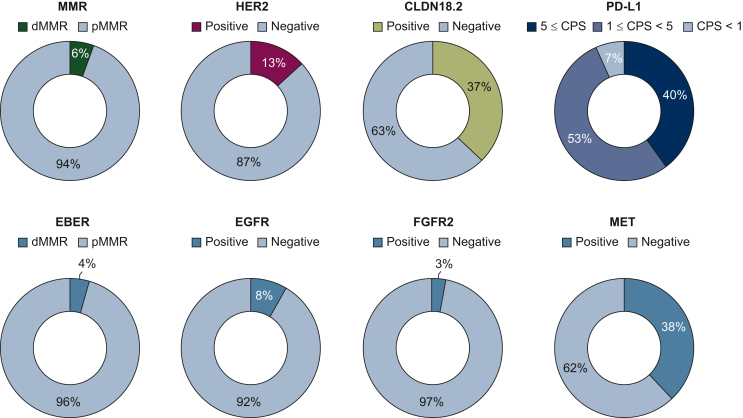
**Biomarker results.** Pie chart depicting the results of the eight biomarkers (MMR, HER2, CLDN18.2, PD-L1, EBER, EGFR, FGFR2, and MET), each showing their respective proportions. CLDN18.2, claudin 18.2; CPS, combined positive score; dMMR, deficient mismatch repair; EBER, Epstein–Barr virus-encoded RNA; EGFR, epidermal growth factor receptor; FGFR2, fibroblast growth factor receptor 2; HER2, human epidermal growth factor receptor 2; MET, mesenchymal epithelial transition; MMR, mismatch repair; PD-L1, programed death-ligand 1; pMMR, MMR proficient.

**Figure 3:**
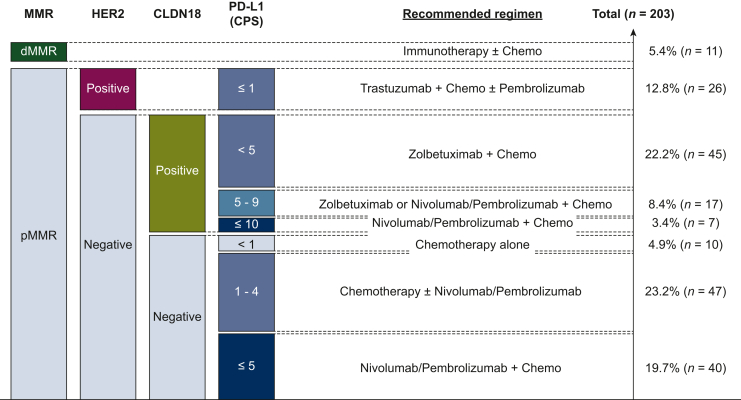
**Suggestionof patient selection according to biomarker status (CPS 5)**. The four bar graphs represent the proportions of each biomarker. Their relative positions are arranged considering the degree of overlap. Each treatment group is accompanied by a description of the recommended therapy tailored to the respective population.

Biomarker status was depicted in Figure 2. dMMR was detected in 11 patients (5.4%). PD-L1 expression was examined using the 22C3 pharm Dx assay in 39 cases (19.2%), 28-8 pharm Dx assay in 143 cases (70.4%) and both in 21 (10.3%). PD-L1 CPS ≥1, ≥5, and ≥10 was observed in 189 (93.1%), 81 (39.9%), and 29 (14.3%) patients, respectively. Among 11 patients with dMMR mGEA, 8 patients had PD-L1 CPS ≥5 tumor but there were some overlaps with HER2 positivity (n = 1) and CLDN18.2 positivity (n = 2). HER2-positivity was confirmed in 27 patients (13.3%), all of whom had CPS ≥1. CLDN18.2 positivity was 37.4% (*n* = 76) in all 203 patients. Double positivity for HER2 and CLDN18.2 was observed in four patients. In the CLDN18.2-positive group (*n* = 75), PD-L1 status was CPS ≥1 in 71 (94.7%), CPS ≥5 in 27 (36.0%), and CPS ≥10 in 8 patients (10.7%), respectively. Meanwhile, among 128 patients with CLDN18.2-negative mGEA, PD-L1 expression was CPS ≥1 in 118 (92.2%), CPS ≥5 in 54 (42.2%) and CPS ≥10 in 21 patients (16.4%), respectively. In consideration with these overlaps, classification of patients with mGEA will become quite subdivided and complicated. We suggested possible stratification for current treatment options as shown in Figure 3 and Supplementary Figure S3, available at https://doi.org/10.1016/j.esmogo.2024.100086. Furthermore, results of other biomarkers were also available in all patients. EBV-positivity was identified in nine patients (4.4%). In addition, the proportion of EGFR-positive, FGFR2-positive, and MET-positive patients was 8.3% (*n* = 17), 3.0% (*n* = 6), and 37.9% (*n* = 77), respectively.

### Transition to first-line chemotherapy

First-line therapy was initiated after determining the biomarker status in 179 patients (88.2%). The remaining patients received chemotherapy before obtaining biomarker results, primarily due to tumor-related symptoms that necessitated urgent chemotherapy. Among 179 patients, 106 patients (59.2%) were enrolled into clinical trials with 45 patients (25.1%) for biomarker-selected studies.

## Discussion

This study underscores the crucial role of comprehensive biomarkers testing in guiding first-line therapy for mGEA, demonstrating its feasibility and efficacy in a clinical setting. Our protocol enabled the successful implementation of upfront testing for key actionable biomarkers like HER2, MMR, PD-L1 CPS, and CLDN18.2, achieving biomarker results within a median TAT of 7 days. This promptness is crucial for timely therapeutic decisions, highlighting the practicality of this approach in everyday clinical practice. Notably, ∼90% of patients could receive treatment after confirming biomarker results. Taking disease progression into consideration, we initiated treatment even in the absence of detectable biomarkers, ensuring that patients did not miss the opportunity for timely intervention.

The biomarker results in this analysis were mostly comparable to those of pivotal studies, supporting their validity in a real-world context. For instance, the HER2 positivity and dMMR rate in our cohort were within the expected range for mGEA.^4^^,^^9^^,^^15^ However, PD-L1 CPS results varied more across studies, reflecting the intrinsic challenges of tumor heterogeneity, assay vulnerability, and inter-pathologist discordance.^16^^,^^17^ Despite these challenges, our data on PD-L1 CPS ≥5 (39.9%) compared similarly to other observational studies, albeit lower than CheckMate-649 (60.4%).^8^^,^^18^ CLDN18.2 positivity was aligned with pivotal trials, further confirming the relevance of our testing in a real-world setting.^19^

We successfully achieved a 100% success rate in conducting multiple biomarkers testing in eligible cases, managing extensive testing of eight biomarkers using 11 FFPE slides in over 90% of cases. Notably, type 4 gastric cancer presents unique challenges due to its tendency to yield biopsy-negative results, a consequence of minimal superficial changes, a sparse distribution of tumor cells, and a predominance of stromal tissue. At our institution, we routinely collect at least six biopsy specimens during initial endoscopy, adhering to current guideline recommendations.^2^ In response to the growing need for comprehensive genomic analyses, we have recently increased the number of biopsy specimens collected to nine. This adjustment accommodates the additional biomarker analysis required by numerous clinical trials, which often stipulate central biomarker assessments.

Referring to hazard ratio for death in previous pivotal trials, we have proposed the recommended regimen for subgroups stratified by biomarker status as illustrated in Figure 3 or Supplementary Figure S3, available at https://doi.org/10.1016/j.esmogo.2024.100086.^9^^,^^10^ Our findings demonstrate that multiple biomarkers testing can guide patient stratification and facilitate tailored treatment based on currently available actionable biomarkers such as HER2, MMR, PD-L1 CPS, and CLDN18.2, enhancing precision therapy. For pMMR, HER2-negative, and CLDN18.2-positive mGEA, zolbetuximab and nivolumab/pembrolizumab are viable therapeutic options. There is no consensus on the optimal cut-off value for CPS. In addition, different toxicity profiles should be considered when choosing treatments. The preferred regimen in first-line therapy may also be influenced by the therapeutic options available in salvage line. While our suggestion is not universally applicable, it underscores the need for a broad adoption of biomarker-driven strategies in the treatment of mGEA and highlights the need for institutional frameworks that enable rapid and precise biomarkers testing.

Looking beyond currently actionable biomarkers, our platform also incorporates emerging targets such as FGFR2, MET, EGFR, and EBV, in an exploratory manner, maintaining high success rates across these tests. However, challenges remain in these settings if there is limited pathologist support, where alternative strategies like transcriptome analysis of circulating DNA, transcriptome analysis, and artificial intelligence (AI)-driven molecular diagnostics could play transformative roles in future personalized medicine.^14^^,^^20^

The major limitation of this study was the retrospective, single-center design. Our institute is a tertiary cancer-specific hospital in Japan. We could carry out endoscopy soon after the first patient visit and IHC testing at our own institution. Multidisciplinary collaboration with medical oncologists, endoscopists and pathologists are indispensable for implementation of biomarker-driven medicine. The evaluation was done by only two board-certificated pathologists (NS and TK). However, local assessments of HER2, MMR, PD-L1, and CLDN18.2 were comparable with those in previous studies.

In conclusion, our experience affirms that multitarget biomarkers testing is not only feasible but also instrumental in guiding first-line treatment for mGEA. Establishing robust institutional protocols and consensus on biomarkers testing is essential for optimizing patient care. Future advancements in technologies such as liquid biopsies and AI promise to further enhance the personalization of cancer therapy.

## Supplementary Data

Supplementary Figure S1 Patients flow

Supplementary Figure S2 Success rate at the first biopsy

Supplementary Figure S3 Suggestion of patient selection according to biomarker status (CPS 1 and 10)
