# Supplementary figures and images for: Clinical implementation of simultaneous multiple biomarkers testing for metastatic or recurrent gastroesophageal adenocarcinoma: a single-institutional experience
Source: ESMO Gastrointest Oncol. 2024 Aug 2;5:100086. doi: 10.1016/j.esmogo.2024.100086 (PMC12836672; doi:10.1016/j.esmogo.2024.100086)

Supple Fig 1.

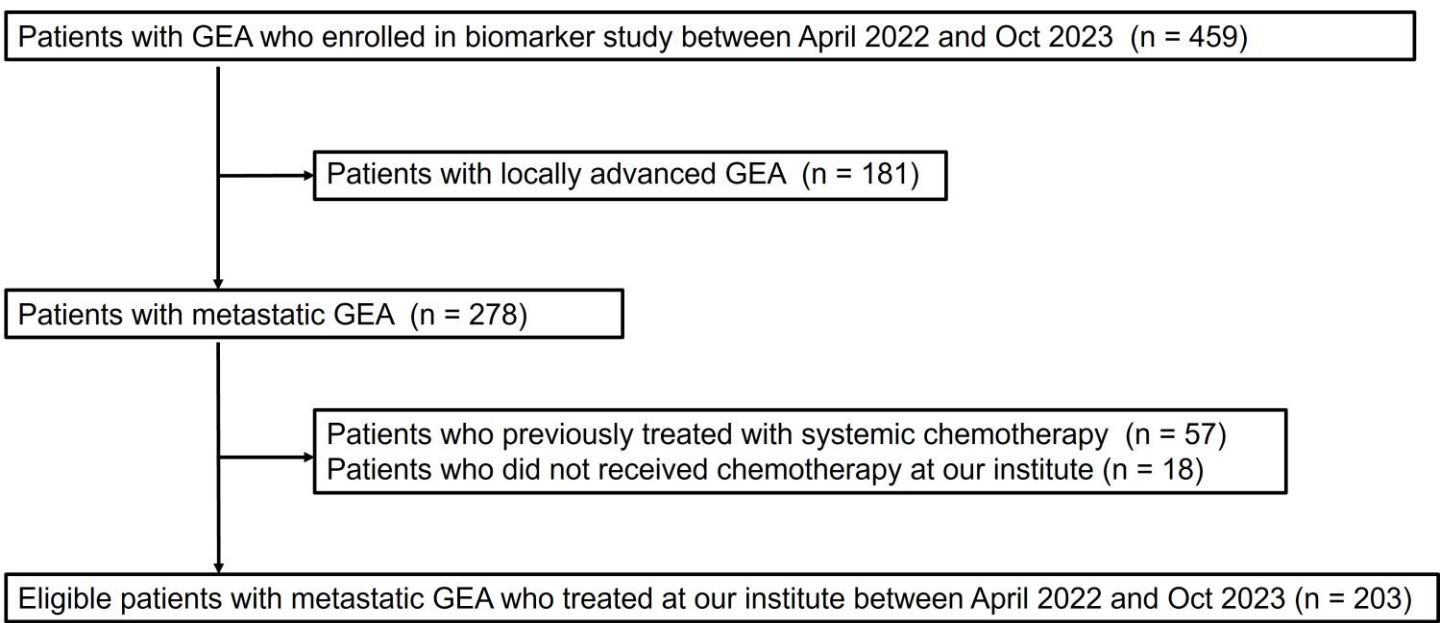

Supplement: Supplementary Figure S1 — Patients flow [file mmc1.pdf]

## Slide 1
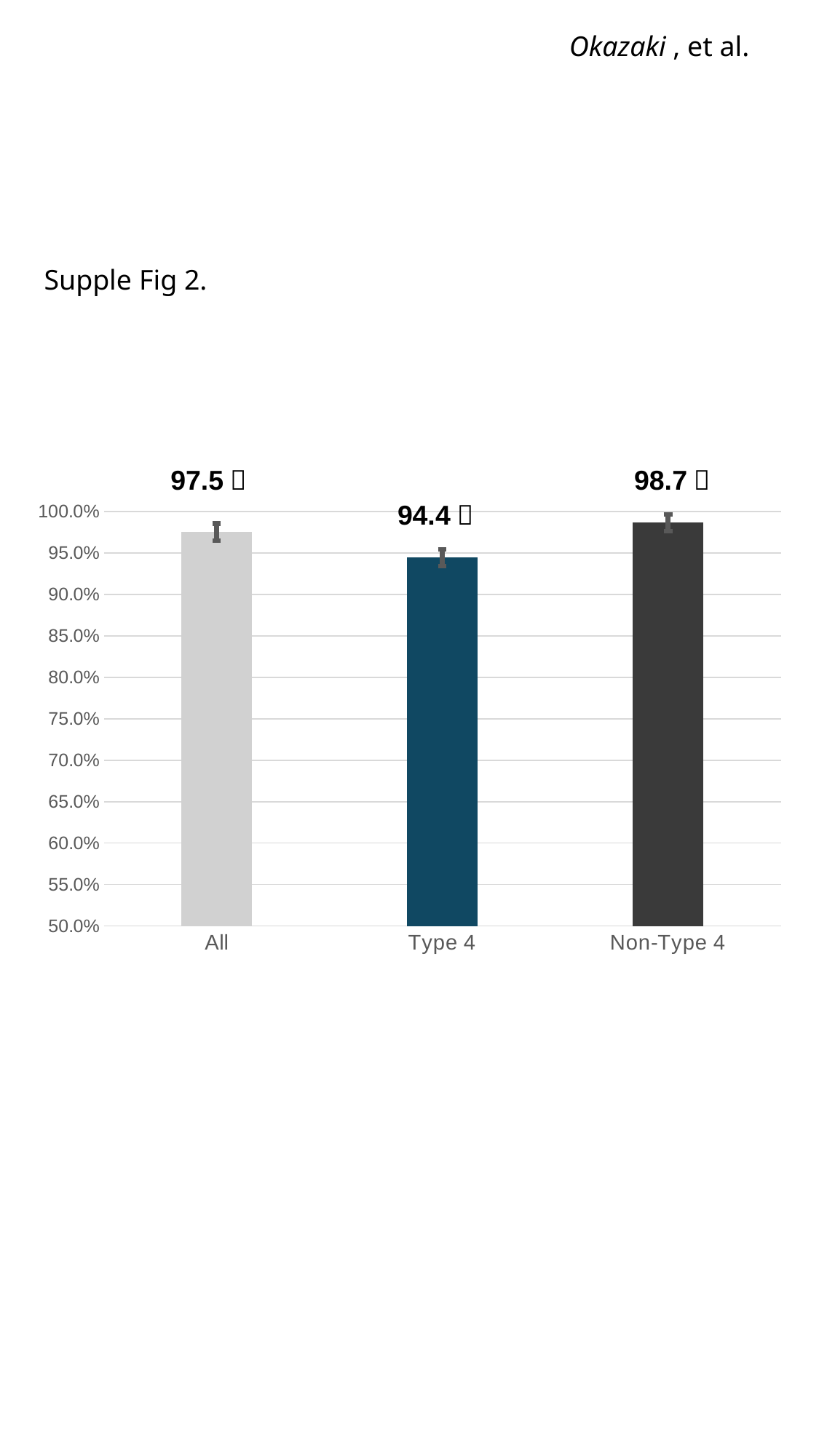

Okazaki , et al.
Supple Fig 2.
97.5％
98.7％
### Chart
| Category | 成功割合 |
|---|---|
| All | 0.9753694581280788 |
| Type 4 | 0.9444444444444444 |
| Non-Type 4 | 0.9865771812080537 |94.4％

Supplement: Supplementary Figure S2 — Success rate at the first biopsy [file mmc2.pptx]

Supple Fig 3.

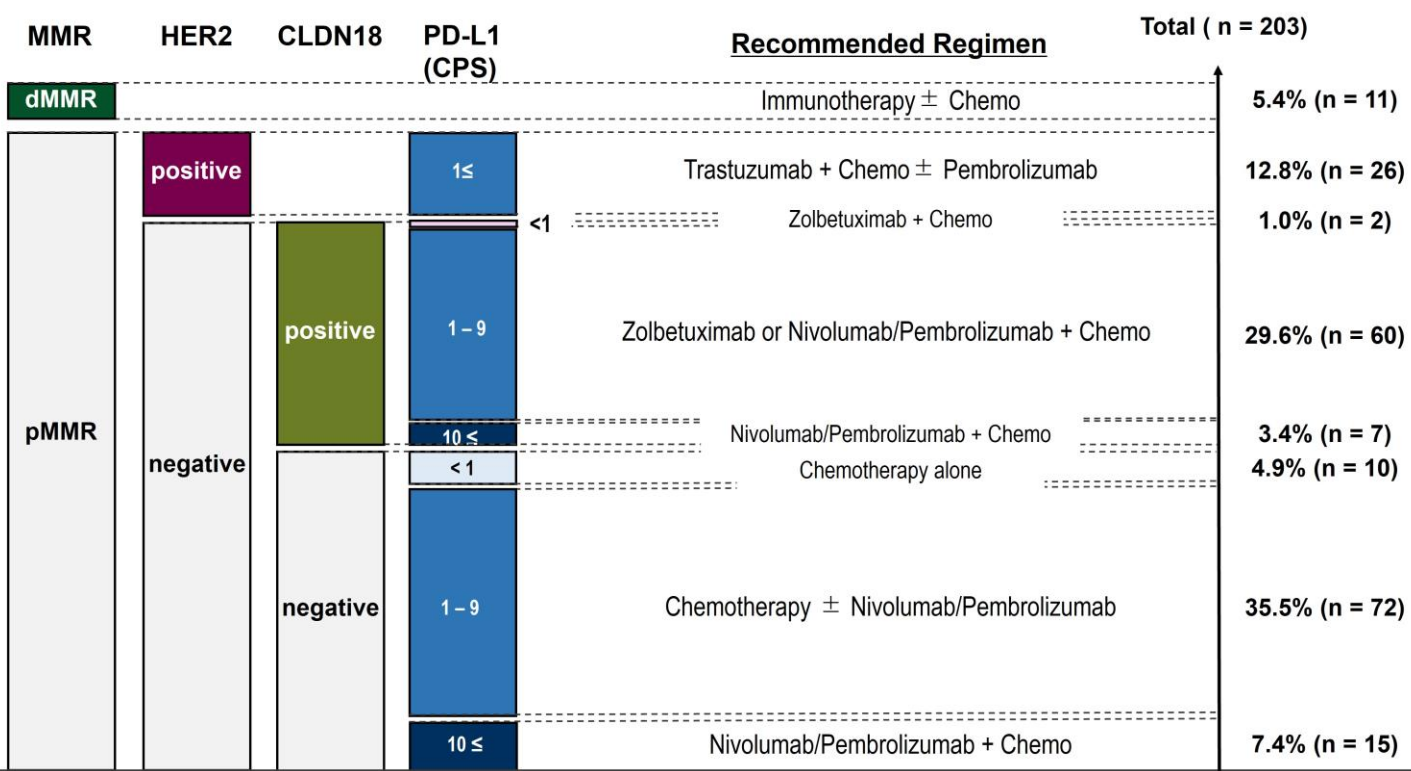

Supplement: Supplementary Figure S3 — Suggestion of patient selection according to biomarker status (CPS 1 and 10) [file mmc3.pdf]
